# Functional Analysis of the Fusion and Attachment Glycoproteins of Mojiang Henipavirus

**DOI:** 10.3390/v13030517

**Published:** 2021-03-22

**Authors:** Sofia Cheliout Da Silva, Lianying Yan, Ha V. Dang, Kai Xu, Jonathan H. Epstein, David Veesler, Christopher C. Broder

**Affiliations:** 1 Department of Microbiology, Uniformed Services University, Bethesda, MD 20814, USA; sofia.dasilva@hhs.gov (S.C.D.S.); lianying.yan.ctr@usuhs.edu (L.Y.); 2 Henry M. Jackson Foundation for the Advancement of Military Medicine, Bethesda, MD 20814, USA; 3 Department of Biochemistry, University of Washington, Seattle, WA 98195, USA; hvdang@uw.edu (H.V.D.); dveesler@uw.edu (D.V.); 4 Vaccine Research Center, National Institutes of Allergy and Infectious Disease, National Institutes of Health, Bethesda, MD 20892, USA; xukai99@gmail.com; 5 EcoHealth Alliance, New York, NY 10001, USA; epstein@ecohealthalliance.org

**Keywords:** mojiang virus, Cedar virus, paramyxoviridae, henipavirus, ephrin ligand, receptor tropism, envelope glycoprotein, nano luciferase, heptad repeat, membrane fusion

## Abstract

Mojiang virus (MojV) is the first henipavirus identified in a rodent and known only by sequence data, whereas all other henipaviruses have been isolated from bats (Hendra virus, Nipah virus, Cedar virus) or discovered by sequence data from material of bat origin (Ghana virus). Ephrin-B2 and -B3 are entry receptors for Hendra and Nipah viruses, but Cedar virus can utilize human ephrin-B1, -B2, -A2 and -A5 and mouse ephrin-A1. However, the entry receptor for MojV remains unknown, and its species tropism is not well characterized. Here, we utilized recombinant full-length and soluble forms of the MojV fusion (F) and attachment (G) glycoproteins in membrane fusion and receptor tropism studies. MojV F and G were functionally competent and mediated cell–cell fusion in primate and rattine cells, albeit with low levels and slow fusion kinetics. Although a relative instability of the pre-fusion conformation of a soluble form of MojV F was observed, MojV F displayed significantly greater fusion activity when heterotypically paired with Ghana virus G. An exhaustive investigation of A- and B-class ephrins indicated that none serve as a primary receptor for MojV. The MojV cell fusion phenotype is therefore likely the result of receptor restriction rather than functional defects in recombinant MojV F and G glycoproteins.

## 1. Introduction

Henipaviruses are unique members of the *Paramyxoviridae* family [1]. The prototypical henipavirus species, Hendra virus (HeV) and Nipah virus (NiV), are highly pathogenic Biological Safety Level-4 (BSL-4) select agents that emerged in the 1990s in Australia and peninsular Malaysia, respectively [2]. They possess a broad host range spanning six mammalian orders [3,4,5] and cause infections that can result in severe respiratory illnesses and/or encephalitis with associated high fatality rates in humans (40–100%) [6,7] and other mammals, such as horses and pigs [3,4,5]. Both HeV and NiV periodically spill over from their bat reservoir hosts. NiV outbreaks are also associated with human-to-human infection transmission and have occurred in the Bangladesh/West Bengal region dubbed the “Nipah belt” on a near annual basis [8,9]. More recent instances of human NiV infection have occurred on the island of Mindanao in the Philippines in 2014 [10] and in the Malabar coast state of Kerala, India in 2018 [11]. However, although there is now a licensed vaccine against HeV infection for horses in Australia, no NiV and HeV therapeutics or vaccines approved for human use are currently available [12,13,14].

The genus *Henipavirus* also includes three additional species, two of which include viruses that were detected in or isolated from individual bats. The species, *Ghanaian bat henipavirus*, includes Ghana virus (GhV), which was identified by targeted RNA sequencing of fecal samples collected from straw-colored fruit bats (*Eidolon helvum*) [15]. Cedar virus (CedV) (*Cedar henipavirus*), a non-pathogenic virus, was isolated from fruit bat urine samples in Australia [16]. The third *Henipavirus* species, Mojiang virus (MojV) (*Mojiang henipavirus*), was discovered in 2012 specimens collected from yellow-breasted rats (*Rattus flavipectus*) in the Tongguan mine in Mojiang, Yunnan, China, where three miners had died of pneumonia of unknown etiology [17]. The clinical presentation of these infections and the confirmed exposure of the miners to horseshoe bats (genus *Rinolophus*), which are known to harbor SARS-like coronaviruses, have recently led to the suspicion, retrospectively, that these cases of pneumonia may be linked to SARS-like coronavirus infection [18]. No viral isolates of GhV and MojV have been recovered to date; GhV and MojV are known only from genetic sequence data [17,19], and the pathogenic potential of either of these henipaviruses remains unknown.

Host cell infection by henipaviruses HeV, NiV and CedV is mediated by their tetrameric attachment glycoprotein (G) and trimeric fusion glycoprotein (F) (reviewed in [20,21,22]). The G glycoprotein is expressed fully functional at the cell surface, whereas the F glycoprotein is initially expressed as an F_0_ inactive precursor, then recycled and cleaved by the endosomal protease cathepsin L to generate a biologically active protein composed of two subunits, F_1_ and F_2_, covalently linked by a disulfide bond and subsequently trafficked back to the plasma membrane [23]. Binding of the G glycoprotein to an entry receptor triggers the F glycoprotein to undergo an irreversible conformational change that mediates the merging of the virion and host cell membranes (reviewed in [24,25]). The F and G glycoprotein-mediated membrane fusion is also responsible for the formation of multi-nucleated giant cells termed syncytia, a hallmark of cytopathic effects (CPE) observed in paramyxovirus infections [26].

Ephrin ligands, which engage in bidirectional signaling with Eph-receptors to mediate cell–cell repulsion, adhesion and attraction mechanisms involved in vascular and neural development, plasticity and repair (reviewed in [27,28]), have been identified as entry receptors of henipaviruses [29,30,31]. These ligands are conserved across mammalian species and classified into two groups: the A-class ephrins (A1 through A5) are tethered to the plasma membrane by a glycosylphosphatidylinositol (GPI) anchor, whereas the B-class ephrins (B1 through B3) are transmembrane proteins with a short cytoplasmic tail [28,32]. The ephrin-B2 and -B3 ligands act as entry receptors for HeV and NiV [33,34,35] through interactions between the binding cleft of the globular head of G and a specific domain of ephrin ligands termed the G-H loop [29,32,36]. CedV, however, is characterized by a uniquely broad ephrin tropism and can utilize mouse ephrin-A1, as well as human ephrin-A2, -A5, -B1 and -B2 as entry receptors [31], whereas GhV G solely recognizes ephrin-B2 as its receptor [30,37,38]. MojV G, conversely, is unable to utilize the canonical ephrin-B2 or -B3 henipavirus receptors to mediate cell–cell fusion; moreover, MojV G does not bind the morbillivirus receptor CD150, nor does it bear sialic acid binding residues [39]. However, the ability of MojV F and G to mediate cell fusion by utilizing any other ephrin ligands is unknown. MojV species cell fusion tropism may be defined, at least in part, by receptor restrictions. Pteropid bat species are the natural reservoir of HeV, NiV, and CedV and Eidolon helvum is the putative reservoir for GhV [16,19,40,41]. Indeed, the ability of CedV G to functionally interact with multiple ephrins including murine ephrin-A1 [31], the fact that MojV is the first henipavirus identified in a rodent [17], and reports of productive experimental HeV and NiV infections in hamsters and guinea pigs [3,4,5], suggests that henipaviruses may naturally infect rodent hosts, perhaps through engagement with other ephrin ligands or rodent-specific ephrin ligands.

In this study, we sought to characterize the functions of the MojV envelope glycoproteins, and we investigated MojV cell fusion species tropism and potential use of both A- and B-class ephrin ligands as fusion triggering receptors. We found that human, non-human primate and rodent cells are susceptible to cell–cell fusion mediated by MojV F and G glycoproteins with a low and slow fusion phenotype. This cell fusion phenotype was also observed with target cells expressing mouse ephrin-A1, rat ephrin-A4 and human ephrin-A5 ligands. Investigation of the basis for the MojV membrane fusion phenotype using an in vitro triggering assay with soluble MojV F (sF) revealed an instability of its pre-fusion conformation. However, a significantly greater cell fusion activity of MojV F was observed when heterotypically paired with GhV G. Taken together, these data indicate that the MojV cell fusion phenotype is likely the result of host cell receptor restrictions rather than inherent functional defects in recombinant MojV F and G.

## 2. Materials and Methods

### 2.1. Cell Lines

BHK-21 cells were obtained from Norman Cooper (National Institute of Health, Bethesda, MD, USA). BSR-T7/5 cells, BHK-21-derived cells that constitutively express T7 polymerase were provided by Tzanko Stantchev (U.S. Food and Drug Administration, Silver Spring, MD, USA). HeLa-USU cells lacking expression of HeV and NiV receptors ephrin-B2 and -B3 were described in previous studies [33]. HEK293T, A549 and Rat2 cells were provided by Brian Schaefer, Regina Day and Chou-Zen Giam (Uniformed Services University, Bethesda, MD, USA), respectively. Vero cells (ATCC CCL-81), CHO-K1 cells (ATCC CCL-61), Neuro-2a cells (ATCC CCL-131), C6 cells (ATCC CCL-107) and L2 cells (ATCC CCL-149) were purchased from the American Type Culture Collection (Manassas, VA, USA). FreeStyle™ 293 cells were obtained from Thermo Fisher Scientific (Waltham, MA, USA). BHK-21, BSR-T7/5, Neuro-2a, HEK293T, HeLa-USU and Vero cells were cultured in Dulbecco’s Modified Eagle’s Medium (Quality Biologicals, Gaithersburg, MD, USA) with 2 mM L-glutamine (Quality Biologicals,), 1% penicillin–streptomycin (Quality Biologicals) and 10% cosmic calf serum (HyClone, Logan, UT, USA) (DMEM-10). A549, CHO-K1 and L2 cells were maintained in Ham’s F-12K (Kaighn’s) Medium (Gibco, Gaithersburg, MD, USA) with 1% penicillin–streptomycin and 10% cosmic calf serum (F-12K-10). C6 cells were grown in Ham’s F-12K (Kaighn’s) Medium supplemented with 1% penicillin–streptomycin, 2.5% cosmic calf serum and 15% horse serum (ATCC 30-2040) (F-12K-2.5/15). FreeStyle™ 293 cells were maintained in Dulbecco’s Modified Eagle’s Medium supplemented with 2 mM L-glutamine, 1% penicillin–streptomycin and 5% cosmic calf serum (DMEM-5). All cell cultures were kept at 37 °C in 5% CO_2_ humidified atmosphere except for Neuro-2a cells and FreeStyle™ 293 cells maintained in 8% CO_2_.

### 2.2. Expression Plasmids

Codon optimized HeV (Genbank NC 001906.3), NiV (GenBank NC 002728.1), CedV (Genbank KP 271122.1), GhV (Genbank NC 025256.1) and MojV (Genbank NC 025352.1) F and G open reading frames (ORFs) were synthesized by GenScript^®^ (Piscataway, NJ, USA) and subcloned into the mammalian expression vector pcDNA3.1 Hygro CMV [42]. To facilitate detection an S peptide tag (S-tag) (KETAAAKFERQHMDS) was added to the end of the cytoplasmic domains of the full-length henipavirus glycoproteins by site-directed mutagenesis (QuickChange II XL, Agilent technologies, Santa Clara, CA, USA). For construction of soluble and secreted versions of F glycoproteins, the C-terminal cytoplasmic and transmembrane domains of CedV F and MojV F were replaced with a GCN4 trimerization motif (GCNt) [43] (MKQIEDKIEEILSKIYHIENEIARIKKLIGE), previously used in the generation of sF trimers of HeV and NiV [44,45], and an S-tag to generate trimeric soluble constructs of CedV sF and MojV sF (Figure S1). To produce tetrameric soluble GhV G (GhV sG) and MojV G (MojV sG), the N-terminal cytoplasmic and transmembrane domains were replaced with an Igк leader sequence (METDTLLLWVLLLWVPGSTGD) and an S-tag followed by a GCN4 tetramerization motif (GCNtet) [43] (MKQIEDKLEEIESKLKKIENELARIKK), as previously described for CedV G (CedV sG) [31] (Figure S2). Expression plasmids encoding genes for human ephrin-A1, -A2, -A3, -A4, -A5, -B1, -B2 and -B3; mouse ephrin-A1; and rat ephrin-A4 were obtained from Origene™ (Rockville, MD, USA). S119P and P122S mutations were introduced in human and rat ephrin-A4, respectively, by site-directed mutagenesis. To create a soluble rat ephrin-A4, the region encoding L26-G171 was subcloned into the Fc tag encoding plasmid BNN-CFC-pcDNA3.1. The Nano luciferase NLuc gene (Genbank LC 167158.1) was codon optimized and synthesized by GenScript^®^ and subcloned under the control of the T7 promoter into a pTM1 provided by Chad Mire (University of Texas Medical Branch, Galveston, TX, USA) (pTM1-NLuc).

### 2.3. Western Blot Analysis

BSR-T7/5 cells grown to 60% confluency were transfected with S-tagged HeV, CedV or MojV F or G constructs or with the empty vector using Lipofectamine™ LTX (Invitrogen, Carlsbad, CA, USA) at a 1:2.1 ratio (µg DNA:µL LTX). After 48 h, lysates were collected in RIPA buffer (Thermo Fisher Scientific) with 1x cOmplete™ Protease Inhibitor Cocktail (Roche, Basel, Switzerland) and incubated with S protein agarose (EMD Biosciences Inc., Madison, WI, USA) at 4 °C overnight. Washed beads were boiled in reducing sample buffer (2x LDS NuPage^®^ sample buffer (Invitrogen), 5% β-mercaptoethanol (Sigma-Aldrich, St Louis, MO, USA)). Proteins were resolved by NuPage^®^ 4–12% Bis-Tris SDS-PAGE (Invitrogen), and detected by Western blot with a horseradish peroxidase (HRP) conjugated anti-S polyclonal rabbit antibody (1:12,500) (Southern Biotechnology, Birmingham, AL, USA).

### 2.4. Syncytium Formation Assay

BSR-T7/5 and BHK-21 cells were grown to 60% confluency and transfected with pcDNA 3.1 Hygro CMV HeV, CedV or MojV F or G alone, or co-transfected with F and G (1:1) or empty vector, as described above. Cells were fixed in methanol and stained with crystal violet between 24 h and 5 days post-transfection. Brightfield microscope images were acquired with a Zeiss Axio Observer A1 inverted microscope (Carl Zeiss Microscopy, Jena, Germany) set to the 20× objective.

### 2.5. Cell–Cell Fusion Nano Luciferase Reporter Gene Assay

Effector BSR-T7/5 cells seeded at 0.6 × 10^5^ cells per well (24-well tissue culture plates) were transfected 24 h after plating with 0.8 µg total DNA. Cells were transfected with pcDNA 3.1 Hygro CMV HeV, CedV or MojV F or G alone, or co-transfected with homologous or heterologous F and G (1:1) HeV, NiV, CedV, GhV or MojV expression plasmid combinations or empty vector. Target HeLa-USU, A549, Vero, BHK-21, CHO-K1, L2, C6 and Rat2 cells grown to 60% confluency were transfected with pTM1-NLuc (1 µg DNA:1 µL Plus™ Reagent:3 µL Lipofectamine™LTX (Invitrogen)). NLuc was chosen as a reporter gene because the Nano luciferase enzyme is more stable and generates 150-fold increased luminescence compared to firefly and renilla luciferases [46,47], allowing for more sensitive detection of low levels of cell–cell fusion. Target HEK293T cells were transfected with pTM1-NLuc using X-tremeGENE™ 9 DNA Transfection Reagent (Roche, Basel, Switzerland) (1 µg DNA:3 µL X-tremeGENE™). In the cell–cell fusion assays assessing ephrin ligand tropism, CHO-K1 cells transfected with pCMV-human, mouse, or rat ephrin expression plasmids, in addition to pTM1-NLuc, were used as target cells instead. All target cells were trypsinized 24 h post-transfection and allowed to recover overnight. Target cells (2.0 × 10^5^ cells per well) were then applied to the effector BSR-T7/5 cell monolayers. Effector/target cells mixtures were harvested 24 h, 48 h, 72 h, 96 h, 120 h, day 6 and day 7 post-application and assayed for luciferase activity in technical triplicates with the Nano-Glo^®^ Luciferase Assay System (Promega, Madison, WI, USA). Data were acquired with a GloMax^®^-Multi+ Detection System plate reader (Promega). Normalized maximum luminescence values (see data analysis) reached by each effector/target cell co-cultures are reported, and corresponding collection times are indicated in each figure.

### 2.6. Data Analysis

Cell–cell fusion reporter gene assays were performed at least three times and in technical triplicates. Relative Luminescence Units (RLUs) from the various cell mixtures were normalized to RLUs from mixtures of each target cell type applied to effector cells transfected with the empty vector (background). Normalized RLUs were compared to background (value of 1) to assess statistical significance by applying Welch’s *t*-test. Statistical analysis was performed with Graphpad Prism. Figures were prepared using Graphpad Prism and Adobe Photoshop.

### 2.7. Protein Cross-Linking Assay

BSR-T7/5 cells transiently expressing full length henipavirus glycoproteins were collected 72 h post-transfection and allowed to recover in DMEM-10 overnight at 37 °C. Cells were washed and re-suspended in 1x PBS pH 7.4 (Quality Biologicals,). Aliquots of 5 × 10^6^ cells were incubated with 0, 1, 3 or 5 mM BS3 (bis(sulfosuccinimidyl)suberate), No-Weigh™ Format (Thermo Fisher Scientific) for 45 min at 4 °C. CedV and MojV sF and CedV, GhV and MojV sG purified proteins (1 µg per sample) were incubated with 0, 0.8, 1.0 and 1.2 mM BS3 for 30 min at room temperature. All reactions were quenched for 15 min at room temperature with 20 mM Tris. Full length proteins in cell lysates were precipitated with S agarose beads as described above. Cross-linked proteins were resolved by SDS-PAGE NuPage^®^ 4–12% Bis-Tris gels for F oligomers, 3–8% Tris-Acetate gels (Invitrogen) for G oligomers and analyzed by Western blot.

### 2.8. Production of Soluble Recombinant Proteins

Previously described methods of production and purification of HeV and NiV sF proteins were applied to obtain the soluble henipavirus glycoproteins used in this study [45]. Briefly, all expression plasmids were transfected with Xtreme Fugene9^®^ (Roche, Basel, Switzerland) into FreeStyle™ 293 cells with exception of the cell line expressing MojV sG which was established in Neuro-2a cells because of an unknown incompatibility of its full-length ectodomain expression in FreeStyle™ 293 cells. To generate cell lines stably expressing the soluble proteins, transfected cells were subjected to drug selection in DMEM-5 containing 50 µg/mL Hygromycin B (Thermo Fisher Scientific) (DMEM-5+). After two rounds of limiting dilution cloning, candidate clones were confirmed by Western blot analysis and expanded in FreeStyle™ 293 Expression Medium (Thermo Fisher Scientific). Clarified supernatants were filtered and subjected to affinity purification on an S protein agarose affinity column (EMD Biosciences, San Diego, CA, USA). MojV sG (Figure S3A), MojV sF (Figure S3B), GhV sG (Figure S3C) and CedV sF (Figure S3D) glycoproteins were eluted, concentrated and purified before being subjected to size exclusion chromatography and analyzed by blue native (BN) PAGE and Western blotting as previously described [42,45]. MojV sG, CedV sG and GhV sG glycoproteins (Figure S2C) and the MojV sF and CedV sF glycoproteins (Figure S1C) were produced in their physiologically relevant oligomeric forms. Additional analysis by negative-staining electron microscopy (nsEM) of the quality of the previously undescribed purified MojV sG, and GhV sG glycoproteins (tetramers) and the MojV sF and CedV sF glycoproteins (trimers) is shown in Figure S4. A FreeStyle™ 293 cell line stably expressing soluble rat ephrin-A4-Fc was created as described above with one modification; the supernatant was purified through a protein G agarose affinity column (EMD Biosciences) and eluted with a 0.1 M Glycine pH 2.5 buffer.

### 2.9. HR2 Peptide Triggering and Capture Assay

Biotinylated peptides corresponding to the heptad repeat 2 (HR2) domain of CedV F (CedV FC2, KVDLSNEINKMNQSLKDSIFYLREAKRILDSVNISL) and MojV F (MojV FC2, KIDIGNQLAGINQTLQEAEDYIEKSEEFLKGVNPSI) were synthesized by New England Peptide™, Inc. (Gardner, MA, USA) and are referred to as FC2 peptides in this study. A henipavirus F glycoprotein heptad-repeat capture assay was adapted from a previously established method [45,48]. Briefly, CedV sF or MojV sF glycoproteins were subjected to a combination of heat (50 °C), trypsin digestion (4 °C) and addition of the corresponding FC2peptide (1:6 ratio; sF:FC2 peptide) in different orders of treatment. The sF/FC2 peptide complexes were incubated for 1 h at 4 °C with a 50% avidin agarose slurry (Vector laboratories, Burlingame, CA, USA) in RIPA buffer and 1× cOmplete™ Protease Inhibitor Cocktail. Reduced samples were analyzed by Western blot with primary mouse anti-MojV F monoclonal antibody (mAb) 4G5 (1:2500), or anti-CedV F polyclonal serum (1:1000) and secondary HRP-conjugated goat anti-mouse IgG (H+L) antibody (1:12,500) (Thermo Fisher Scientific).

### 2.10. Generation of Antibodies against CedV and MojV sF Glycoproteins

Mice studies were conducted in accordance with protocol MIC 16-262 approved by the Uniformed Service University Animal Care and Use Committee. Seven-week-old BALB/cByJ mice (stock#001026) purchased from Jackson Labs (Bar Harbor, ME, USA) were immunized with purified MojV sF (Figure S3B) and CedV sF (Figure S3D) glycoproteins in sterile 1XPBS:MPL-TDM adjuvant and polyclonal and mAbs were produced as previously described [45]. The anti-MojV F mAb 4G5 is conformation independent.

### 2.11. Sequence Analysis

Sequences for human, mouse and rat ephrin ligands were aligned and analyzed with Clone Manager Suite9 software (Scientific & Educational Software, Cary, NC, USA).

### 2.12. Co-Precipitation Assay

Soluble Fc-conjugated A-class and B-class ephrins including human ephrin-A1 Fc (provided by Kai Xu, National Institutes of Health, Bethesda, MD, USA), mouse ephrin-A1, mouse ephrin-A2, human ephrin-A3, human ephrin-A4, human ephrin-A5, mouse ephrin-B1, mouse ephrin-B2 and human ephrin-B3 (R&D Systems; Minneapolis, MN, USA) and rat ephrin-A4 (see above) were incubated with purified MojV sG, GhV sG, and CedV sG proteins at a 1:2 ratio (µg sG:µg ephrin) in RIPA buffer supplemented with 1X cOmplete protease inhibitor at 4 °C overnight. Samples were incubated with protein G agarose slurry or S protein agarose slurry for 2.5 h at 4 °C. Co-precipitated henipavirus sG/ephrin ligand complexes were analyzed by Western blot as described above. Blots were probed with HRP-conjugated anti-S-tag rabbit polyclonal antibody.

### 2.13. Biolayer Interferometry Assay

Mouse ephrin-A1, human ephrin-A4, human ephrin-A5, human ephrin-B2 (R&D Systems) and rat ephrin-A4 (see above) were diluted in kinetics buffer (KB: 1× PBS, 0.001% BSA, 0.02% Tween 20 and 0.005% NaN3 (ForteBio, Fremont, CA, USA)) to 600 nM. CedV sG was diluted to 14 µg/mL and GhV sG and MojV sG were diluted to 21 µg/mL in 10 mM acetate buffer pH 5.0. Assays were performed as previously described [48] with immersion of CedV, GhV and MojV G-loaded AR2G sensors into the different ephrins solutions for 1000s (association phase) and then into KB for 1000s (dissociation phase).

### 2.14. Electron Microscopic Imaging of Negative-Stained sG and sF

Specimen preparation and electron microscopic imaging of negative-stained samples (nsEM) were performed following the conventional negative-staining protocol [49]. Briefly, G and F glycoproteins were diluted to a concentration of approximately 0.02 mg/mL with 10 mM HEPES, pH 7.4, supplemented with 150 mM NaCl. A 4.8-μL drop of the diluted sample was placed on a freshly glow-discharged carbon-coated copper grid. The drop was immediately removed with filter paper, and the grid was washed 3x with the same buffer, followed by negative staining 3× with 0.75% uranyl formate. Datasets were collected using a Thermo Scientific Talos F200C transmission electron microscope operated at 200 kV and equipped with a Ceta camera (Thermo Fisher Scientific). The nominal magnification was 57,000×, corresponding to a pixel size of 2.53 Å, and the defocus was set at −1.2 μm. Data were collected automatically using EPU. Single particle analysis was performed using CryoSPARC.

## 3. Results

### 3.1. Expression of Recombinant Mojiang Virus Attachment and Fusion Glycoproteins

**Figure 1:**
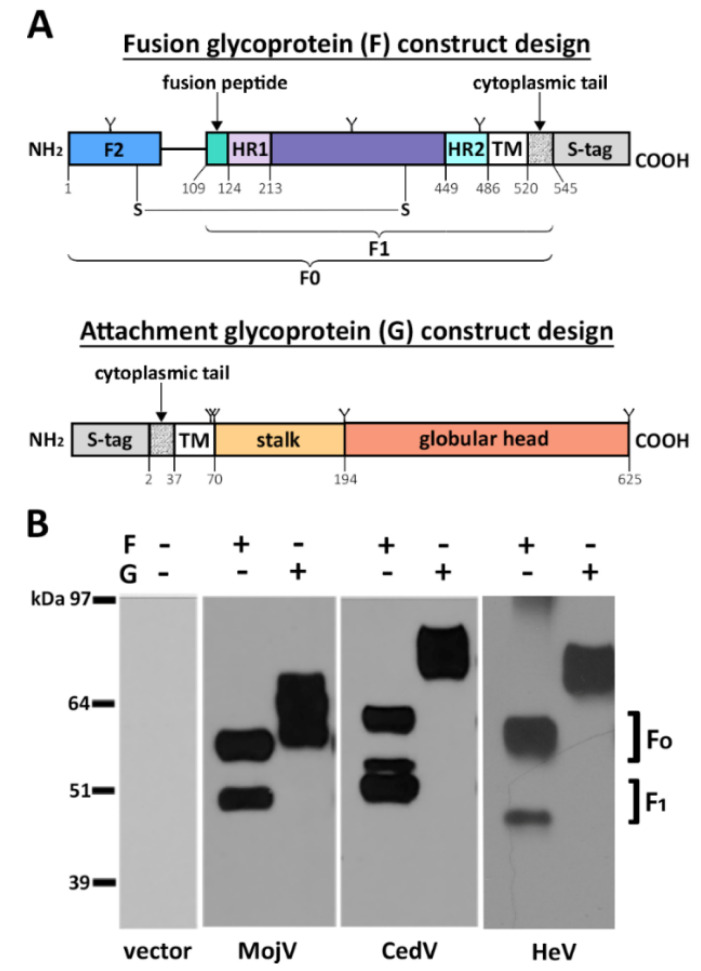
Recombinant Mojiang virus (MojV) fusion (F) and attachment (G) glycoprotein design and expression. (**A**) The open reading frames of MojV attachment (G) and fusion (F) glycoproteins were subcloned into a pcDNA3.1 Hygro CMV expression vector. The constructs were designed to express an S-peptide tag at the cytoplasmic tail end of each glycoprotein, at the C-terminus of F and at the N-terminus of G. Functional domains of the attachment and fusion glycoproteins were predicted using Clustal Omega 1.2.4 sequence alignment [50], SWISS-MODEL homology modelling [51,52,53,54,55] and SABLE secondary structure prediction servers [56,57,58]. F_0_: fusion glycoprotein precursor. F_1_, F_2_: subunits of the mature fusion glycoprotein. TM: transmembrane domain. HR1: heptad repeat 1. HR2: heptad repeat 2. Y: N-glycosylation sites predicted by NetNGlyc 1.0 Server (Technical University of Denmark) at positions 69, 283 and 484 for MojV F and 61, 64, 189 and 619 for MojV G. The disulfide bond between C70 and C370 was predicted by the DISULFIND web server and is depicted as S-S [59]. (**B**) Transient expression of S-tagged MojV, CedV and HeV attachment and fusion glycoproteins in BSR-T7/5 cells. Cell lysates were precipitated with anti-S agarose beads 48 h post transfection. Western blot analysis was performed, and the membranes were probed with rabbit anti-S peptide:HRP antibodies (1:12,500).

MojV F and G glycoprotein expression constructs of codon optimized ORFs were synthesized and included an S-tag at the cytoplasmic end of each protein to facilitate detection (Figure 1A). Recombinant MojV F and G glycoproteins transiently expressed in BSRT7/5 cells displayed gel migration patterns similar to those of HeV and CedV F and G glycoproteins (Figure 1B) and were congruent with previous observations [39].

The MojV, HeV and CedV F glycoproteins all displayed an F_0_/F_1_ band pattern indicating proper cellular proteolytic processing of the F_0_ precursor into the mature F_1_/F_2_ form of the glycoproteins (Figure 1B). Densitometry analysis revealed that ~50% of MojV F_0_, similar to HeV and CedV F_0_, is processed into F_1_/F_2_ (Figure 1B). Because the S-tag is located at the carboxyl-terminus of the F constructs, only the unprocessed F_0_ precursor and the F_1_ subunit are detected. Analysis of MojV G by SDS-PAGE under reducing conditions revealed two major bands with apparent MWs of ~62 to ~64 kDa, ~10 kDa smaller in comparison to HeV G ~74 to 76 kDa [60] and CedV G ~74 to 76 kDa (Figure 1B), likely due to the unusual absence of N-linked glycosylation sites in the β propeller domain of its globular head as previously reported [39].

### 3.2. Functional Assessment of Recombinant MojV F and G Glycoproteins

**Figure 2:**
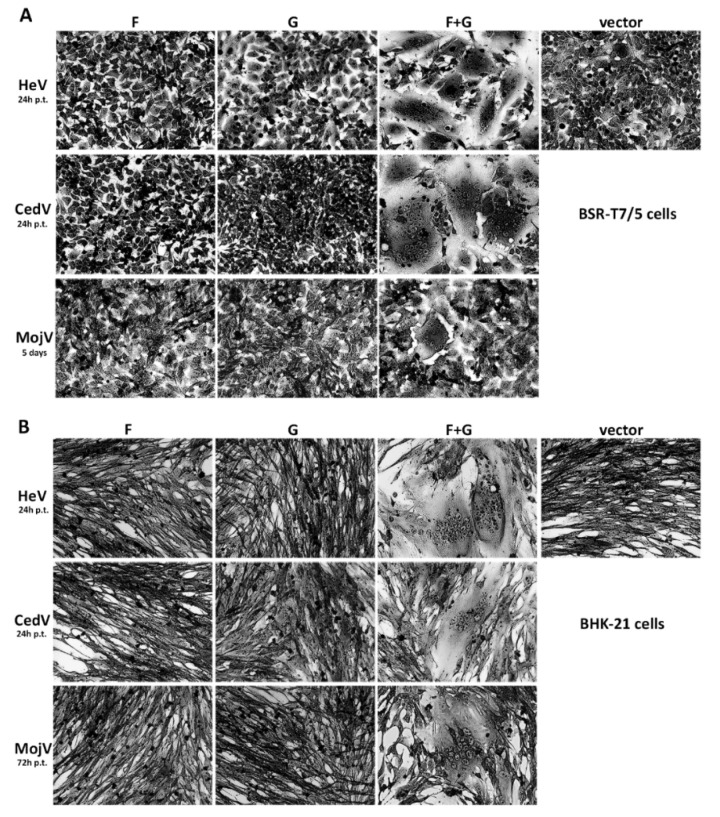
MojV F and G are functional and mediate cell–cell fusion as evidenced by syncytial formation. (**A**) BSR-T7/5 and (**B**) BHK-21 cells were transfected with pcDNA 3.1 Hygro CMV henipavirus F or G alone, or co-transfected with henipavirus F and G or with empty vector. Upon observation of giant multinucleated cell formations (syncytia), the cells were fixed in methanol and stained with crystal violet at the indicated time. Images were obtained with a Zeiss Axio Observer A1 inverted microscope with a 20× objective. p.t.: post-transfection.

The functionality of MojV F and G glycoproteins was qualitatively evaluated in a syncytium formation assay (Figure 2). While syncytia rapidly developed and spread through cell cultures co-expressing HeV F and G or CedV F and G within 24 h post-transfection, syncytia in cells co-expressing MojV F and G were much less extensive and were only observed 5 days post-transfection (Figure 2A). The functionality of MojV F and G was confirmed by repeating this experiment in BHK-21 cells (Figure 2B). The kinetics of syncytia formation in BHK-21 cells co-transfected with MojV F and G differed from those observed in BSR-T7/5 and syncytia were detected 3 days post-co-transfection in BHK-21 cells (Figure 2B). In addition, syncytia formation mediated by MojV F and G was not detected when this experiment was conducted using HEK293T or Vero cells (Figure S5A,B), which are known to be susceptible to HeV, NiV [61] and CedV [31] F and G-mediated cell–cell fusion [39].

### 3.3. Species Susceptibility to MojV F and G Mediated Fusion

**Figure 3:**
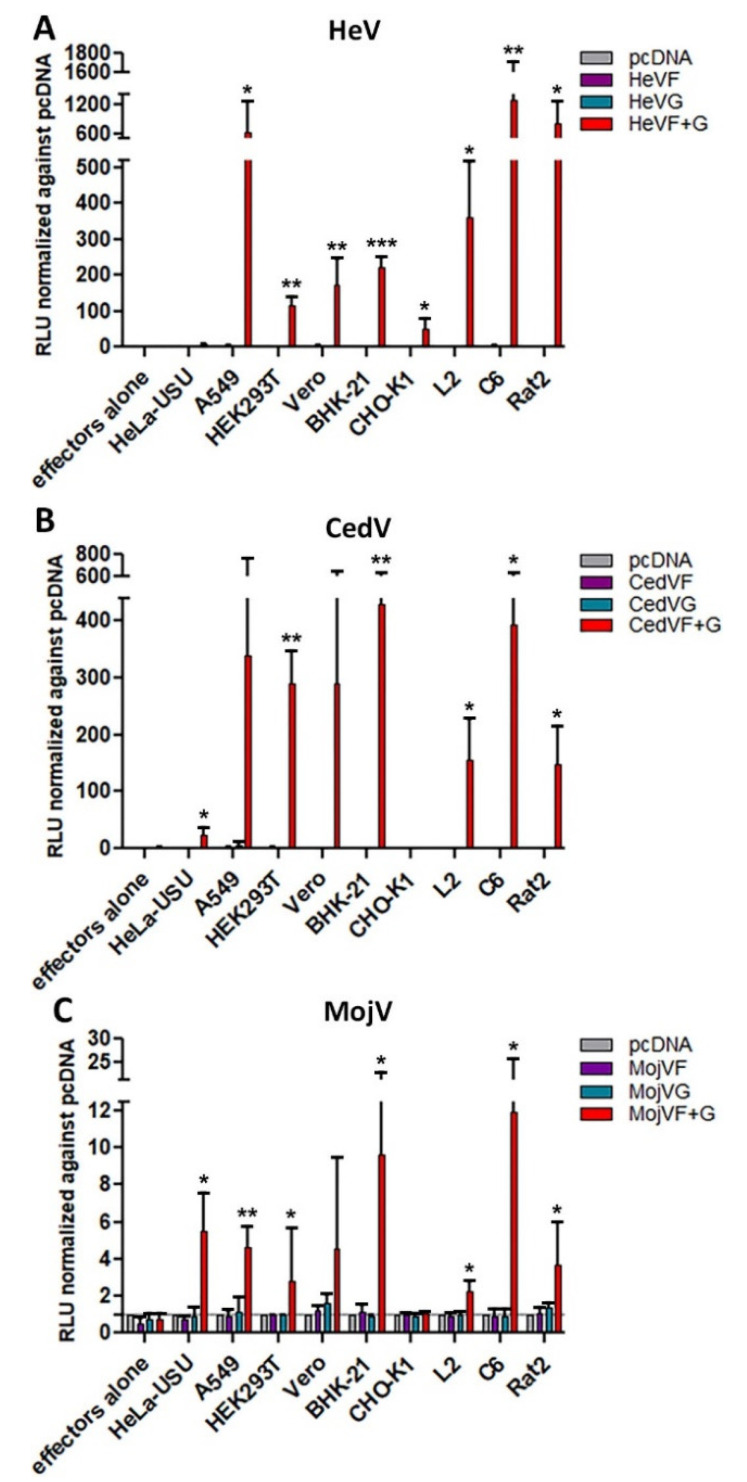
MojV F and G can mediate cell–cell fusion with human, non-human primate and rodent cells in a quantitative Nano luciferase reporter cell–cell fusion assay. BSR-T7/5 effector cells that constitutively express T7 polymerase were transfected with empty vector, henipavirus F alone, henipavirus G alone or co-transfected with henipavirus F and G. Human, non-human primate and rodent target cells transfected with the pTM1-Nluc reporter plasmid were incubated with (**A**) HeV, (**B**) CedV or (**C**) MojV effector cells. Effector/target cells mixtures were harvested 24 h, 48 h, 72 h, 96 h, 120 h, day 6 and day 7 post-application of target cells to effector monolayers and assayed for luciferase activity at the time of harvest. Cell–cell fusion was indirectly measured by recording Relative Luminescence Unit (RLU) upon application of Nano-Glo^®^ substrate. Normalized maximum RLUs detected for each cell line are presented here. Maximum RLUs were recorded 48 h post-application of target cells to effector monolayers co-expressing HeV (**A**) or CedV (**B**) F and G constructs. Effector cells co-transfected with MojV (**C**) F and G constructs yielded maximum RLUs on day 4 with C6 and Rat2 cells; day 5 with HeLa-USU, A549, HEK293T and L2 cells; and day 6 with Vero and BHK-21 cells. Maximum RLUs normalized against values obtained from target cells co-incubated with effector cells transfected with empty vector are reported here. The grey line represents the normalized reference level of 1 (background). Experiments were repeated at least three times in three technical replicates. The error bars represent the standard deviation. Data were analyzed by Welch’s t-test to compare normalized maximum RLUs obtained from each target cell line co-cultured with F and G transfected effector cells to that of target cells co-cultured with effector cells expressing F alone. * *p* ≤ 0.05, ** *p* ≤ 0.01, *** *p* ≤ 0.001.

The susceptibility of mammalian cells to support MojV F and G-mediated membrane fusion was investigated quantitatively using human (HeLa-USU, A549, HEK293T), non-human primate (Vero) and rodent (BHK-21, CHO-K1, L2 rat, C6 rat, Rat2) cells as the target cell population with BSR-T7/5 cells expressing MojV F and G as effector cells or HeV or CedV F and G as positive effector cell controls (Figure 3).

A549, HEK293T, Vero, BHK-21, as well as L2, C6 and Rat2 cells, were permissive to both HeV and CedV F and G-mediated cell–cell fusion (Figure 3A,B). MojV F and G mediated varying levels of cell–cell fusion with human (A549, HEK293T, HeLa-USU), non-human primate (Vero) and rodent (BHK-21, L2, C6, Rat2) cells (Figure 3C). The highest levels of cell–cell fusion measured were observed with C6 rat brain cells (Figure 3C). The Nano luciferase reporter gene cell–cell fusion assay revealed that maximum luminescence levels were reached much faster in those cell cultures containing HeV or CedV F and G expressing effector cells (within 48 h) as compared to cultures containing effector cells expressing MojV F and G (within 4 to 6 days) (Figure 3). Furthermore, the levels of MojV F and G-mediated cell–cell fusion were markedly lower than those generated by HeV or CedV F and G (Figure 3), in agreement with the less extensive syncytial formations observed by microscopy (Figure 2). However, although MojV F and G glycoprotein-mediated cell fusion was less robust and slower to develop, these results demonstrated that additional target cells of human, non-human primate and rodent origin, particularly the rat brain cell line C6, were permissive to MojV F and G glycoprotein-mediated fusion.

### 3.4. Role of A- and B-Class Ephrin Ligands in MojV F and G Mediated Membrane Fusion

MojV was identified through sequencing data obtained from *Rattus flavipectus* samples [17], and since CedV G can utilize mouse ephrin-A1 ligand as a receptor [31] in addition to human ephrin-A2, -A5, -B1 and -B2, the possibility that MojV G may utilize rodent and/or human ephrin ligands other than ephrin-B2 and -B3, which MojV G does not recognize [39], was examined. Co-precipitation assays were performed to assess protein/protein interaction between a soluble tetrameric form of MojV G (MojV sG) (Figure S4A) and a panel of A- and B-class ephrin ligands, and included soluble tetrameric CedV G (CedV sG) [31] and soluble tetrameric GhV G (GhV sG) (Figure S4C) glycoproteins as controls. The results from these co-precipitation assays with CedV sG were congruous with the observed cell fusion mediated by CedV F and G with cells expressing mouse ephrin-A1; human ephrin-A2, -A5, -B1 and -B2 [31]; and CedV G co-precipitated with soluble mouse ephrin-A1, -A2, -B1 and -B2; and human ephrin-A5 (Figure S2F) [31]. GhV sG co-precipitated only with ephrin-B2 (Figure S2F,G) in agreement with previous reports [30,37,38]. No interactions between MojV sG and any of the ephrin ligands tested were detected by co-precipitation (Figure S2F,G). However, because such low and slow MojV F and G-mediated cell fusion was recorded in the syncytia (Figure 2) and Nano luciferase assays (Figure 3C), the possibility that weak or transient interactions between MojV G and an ephrin ligand capable of triggering F glycoprotein-mediated fusion, yet not measurable by co-precipitation, could not be excluded. Therefore, a panel of A- and B-class ephrin ligands was tested using the Nano luciferase reporter cell fusion assay with BSR-T7/5 effector cells co-expressing MojV F and G and target cells expressing individual ephrin ligands.

Sequence alignment of the G-H loop of all mouse and human ephrin ligands revealed differences only between mouse and human ephrin-A1 [31] and ephrin-A4. It was previously demonstrated that CedV can utilize mouse ephrin-A1 but not human ephrin-A1, which differs by 1 residue at position 115 within the critical G-H loop region [31]. This single amino acid difference was responsible for supporting CedV F and G-mediated fusion [31]. Additionally, amino acid residues in two ephrin G-H loop positions important for binding to henipavirus G glycoproteins were noted when comparing mouse to human ephrin-A4 sequences. Here, human ephrin-A4 F115 is replaced by Y118 in mouse ephrin-A4, and both are hydrophobic aromatic residues; this substitution would presumably not significantly impact binding to a henipavirus G glycoprotein, whereas nucleophilic residue S119 in human ephrin-A4 is replaced by hydrophobic residue P122 in mouse ephrin-A4. Further, the only difference noted between critical residues within the human and rat G-H loop sequences across all eight A- and B-class ephrin ligands was the same P residue. Therefore, mouse ephrin-A1 and rat ephrin-A4 were included in the otherwise all human ephrin ligand panel examined by the Nano luciferase cell–cell fusion assay.

**Figure 4:**
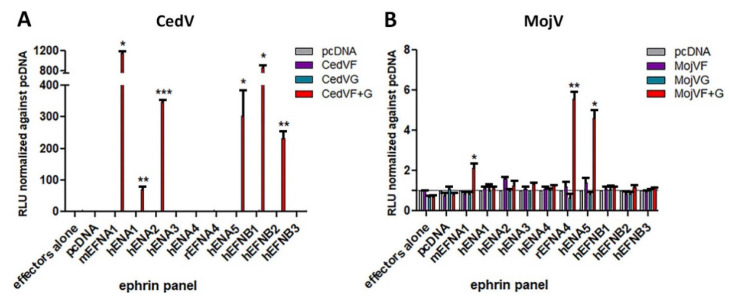
Weak MojV F and G-mediated cell–cell fusion is detected with cells expressing human and rodent A-class ephrin ligands. Cell–cell fusion reporter assays were performed as described in Figure 3 with the following modifications. CHO-K1 target cells were co-transfected with the pTM1-Nluc reporter plasmid and one ephrin-expressing plasmid at a time or an empty vector (pcDNA). Cell mixtures of effector and target cells were assayed for Nano luciferase activity. (**A**) CedV F and G or (**B**) MojV F and G effector cells were tested for their ability to mediate cell–cell fusion against a panel of target cells transiently expressing human and rodent ephrin ligands. Maximum RLUs were recorded 48h post-application of target cells to effector monolayers co-expressing CedV F and G constructs. Co-cultures containing effector cells co-transfected with MojV F and G constructs yielded maximum RLUs on day 6. The grey line represents the normalized reference level of 1 (background). The experiments were performed at least 3 times in technical triplicates. m: mouse ephrin ligand; h: human ephrin ligand; r: rat ephrin ligand. Data were analyzed by Welch’s *t* test comparing the normalized maximum RLU means of F and G expressing effector cells co-cultured with target cells transfected with an ephrin construct to effector cells co-cultured with target cells transfected with an empty vector (pcDNA). The error bars represent the standard deviation. * *p* ≤ 0.05, ** *p* ≤ 0.01, *** *p* ≤ 0.001.

CHO-K1 cells were chosen as target cells because they are non-permissive to MojV and CedV F and G-mediated cell–cell fusion (Figure 3B,C) [31]. CedV F and G expressing effector cells were used as a control and the resulting cell fusion data were congruent with earlier findings showing permissive fusion with mouse ephrin-A1 and human ephrin-A2, -A5, -B1 and –B2 (Figure 4A) [31]. MojV F and G-mediated cell fusion was detected with target cells expressing rat ephrin-A4, human ephrin-A5 and mouse ephrin-A1, but again at much lower levels and slower kinetics (Figure 4B).

**Figure 5:**
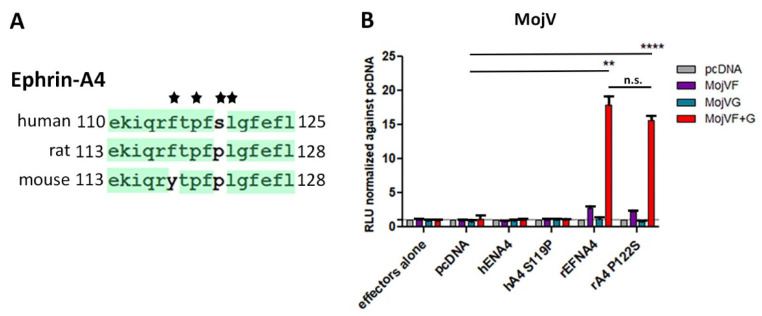
Contribution of rat ephrin-A4 residue P122 to functional interactions with MojV G. (**A**) Sequence alignment of critical residues (*) of the G-H loop of human, rat and mouse ephrin-A4 ligand. (**B**) Cell–cell fusion Nano luciferase assay assessing MojV F and G-mediated fusion with CHO-K1 target cells transiently expressing wild-type or mutant human or rat ephrin ligand A4. m: mouse ephrin ligand; h: human ephrin ligand; r: rat ephrin ligand. Co-cultures containing effector cells co-transfected with MojV F and G constructs yielded maximum RLUs on day 6. The experiments were performed at least 3 times in technical triplicates. A representative experiment is shown here. Data were analyzed by Welch’s *t* test as described in Figure 4. The error bars represent the standard deviation. ** *p* ≤ 0.01, **** *p* ≤ 0.0001. n.s. not significant.

To further analyze a role of the differing amino acid residue P122/S119 in rat ephrin-A4 versus human ephrin-A4 in supporting this low level of cell fusion, a mutant human ephrin-A4 with a G-H loop sequence mimicking the rat ephrin-A4 G-H loop (hEFNA4 S119P) and the converse mutant in rat ephrin-A4 (rEFNA4 P122S) were generated (Figure 5A) and tested in the cell fusion assay. However, mimicking the human G-H loop in the context of the rat ephrin-A4 protein did not abrogate the low and slow level of cell fusion and, introducing the rat G-H loop sequence into the human ephrin-A4 protein did not restore fusion (Figure 5B). There was also no significant difference in MojV F and G-mediated fusion levels with target cells expressing rat ephrin-A4 and rat ephrin-A4 P122S (Figure 5B). In addition, co-precipitation experiments using MojV sG with soluble rat ephrin-A4 (Figure S2D,E) failed to show any binding (Figure S2F,G). Additional Biolayer Interferometry (BLI) assays were performed to assess any possible specific protein/protein interactions; however, no significant binding of MojV sG to rat ephrin-A4, mouse ephrin-A1 or human ephrin-A4, -A5 was detected (Figure S2G). Together, these findings suggest that if A-class ephrin ligands play a role in MojV F and G-mediated cell fusion, they most likely do not act as primary functional receptor(s) for MojV.

### 3.5. Oligomerization Status of MojV F and MojV G

**Figure 6:**
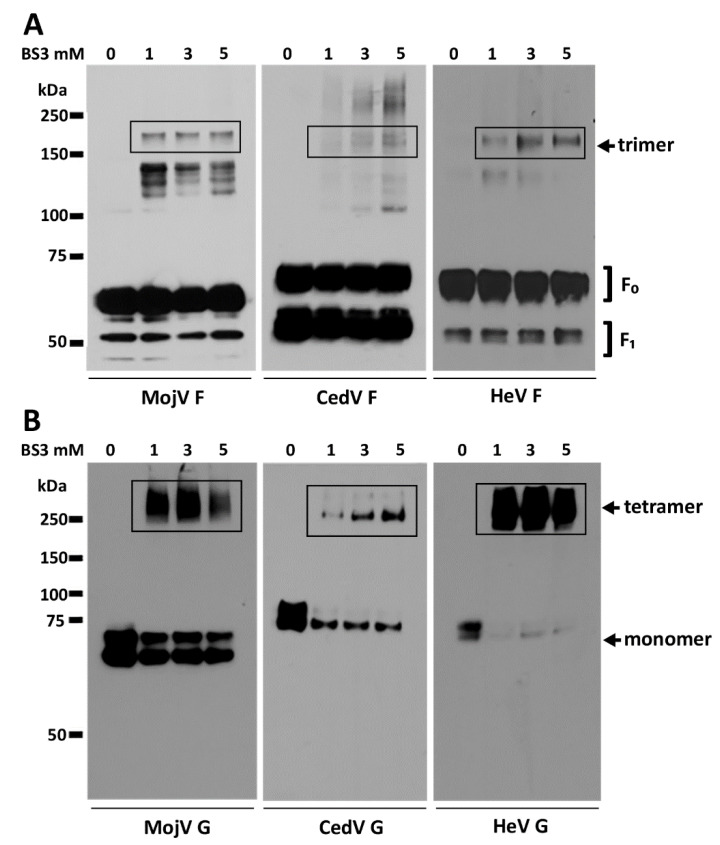
MojV F and G are expressed as trimers and tetramers, respectively, at the cell surface. The oligomerization status of cell-surface-expressed (**A**) MojV F and (**B**) MojV G was assessed by treating cells transfected with each construct with increasing concentrations (0, 1, 3, 5 mM) of the cell-surface protein cross-linking reagent BS3. Cross-linked S-tagged proteins were precipitated with S agarose beads. The precipitates were resolved by SDS-PAGE and analyzed by Western blot under reducing conditions. HeV and CedV recombinant glycoproteins were included as controls.

To investigate whether the low cell fusion levels and slow kinetics observed were the result of major structural defects, the oligomeric status of MojV F and G was evaluated in a protein cross-linking assay using the membrane impermeable reagent bis(sulfosuccinimidyl)suberate (BS3). BSR-T7/5 cells transfected with HeV, CedV and MojV F or G constructs were treated with increasing concentrations of BS3; S-tagged proteins were precipitated and analyzed under reducing conditions by SDS-PAGE and Western blotting (Figure 6). Although the efficiency of cross-linking was poor in this assay, MojV F glycoprotein was detected as an oligomer with an apparent trimeric molecular weight of ~180 kDa similar to CedV F and HeV F (Figure 6A). The HeV, CedV and MojV G glycoproteins were all detected as tetrameric oligomers at the cell surface (Figure 6B).

### 3.6. MojV Soluble F Glycoprotein Pre- to Post-Fusion Conformational Change

**Figure 7:**
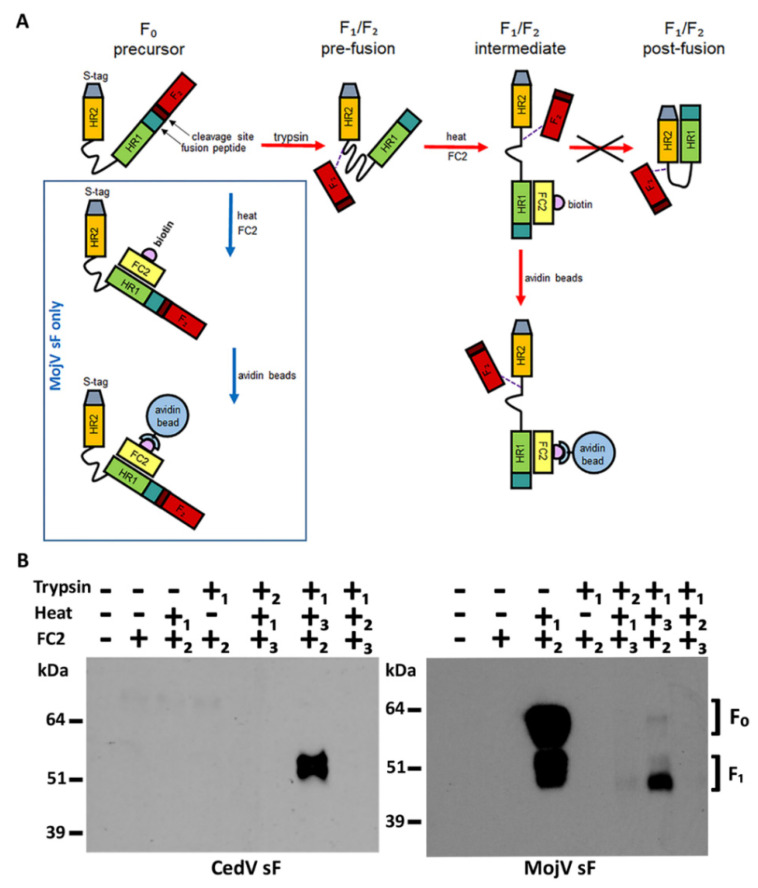
MojV sF can be triggered to undergo a pre- to post-fusion conformational change. (**A**) HR2 heptad peptide triggering and capture assay. The henipavirus sF_0_ is cleaved into the disulfide linked sF_1_ and F_2_ subunits by trypsin digestion. The pre-fusion conformation of cleaved sF trimer is triggered into transitioning from pre- to post-fusion conformation by heat application. Upon triggering, pre-fusion sF rearranges into an intermediate structure before reaching its postfusion conformation. This intermediate conformation allows access to the HR1 domain of sF_1_ by a biotinylated HR2 peptide (FC2). Interaction between intermediate stage sF_1_ and the FC2 peptide prevents sF from completing its transition into the post-fusion conformation by blocking HR1/HR2 interactions. The FC2 peptide thus “captures” an intermediate conformation of sF as it transitions from pre- to post-fusion conformation. The sF/FC2 peptide complex can then be precipitated by incubation with avidin beads. The dashed line represents the disulfide bond between the F_1_ and F_2_ subunits. (**B**) Soluble MojV F (MojV sF) and CedV F (CedV sF) proteins were treated with various combinations of trypsin, heat and the addition of FC2 peptide. The order of treatment applied in each combination in numbered 1, 2, 3. Treated protein complexes were precipitated with avidin agarose beads, resolved under reducing conditions by SDS-PAGE and analyzed by Western blot. CedV sF was detected with mouse polyclonal anti-CedV sF serum at a 1:1000 dilution. MojV sF was detected with mAb 4G5 at a 1:2500 dilution. The F_2_ is not detected. This experiment was performed more than 5 times with different preparations of sF glycoprotein trimers.

The possibility that a defect in the ability of the MojV F glycoprotein to undergo a pre- to post-fusion conformational change required for efficient cell fusion was investigated using a previously developed HR2 heptad peptide triggering and capture assay [45,48]. The F glycoproteins of henipaviruses, upon triggering, adopt an intermediate conformation that establishes a protein bridge between the virion and host cell membranes (reviewed in [24,25]). In this state, the heptad repeat HR1 and HR2 domains of each F_1_ subunit within the F trimer can interact and fold, leading to the formation of a six-helix bundle hairpin (6 HB) structure that promotes the subsequent merging of the virion and cellular membranes and virus infection. Previous studies established that recombinant pre-fusion conformation forms of trimeric soluble HeV F (HeV sF) and NiV F (NiV sF) glycoproteins could be triggered in vitro to refold into post-fusion conformations by application of trypsin and heat [45]. During the conformational transition of sF from its pre- to post-fusion form, if biotinylated HR2 peptides (FC2 peptides) homologous to HeV and NiV F were present, the labeled peptide would bind and capture the sF trimer in an intermediate state between pre- and post-fusion conformations [45]. To establish that MojV F was similarly capable of measurable activation, triggering and transition from a pre- to post-fusion conformation, a soluble construct of MojV F was prepared (MojV sF) along with CedV F (CedV sF) (Figure S1A) as an additional control as previously performed with HeV and NiV sF constructs [44,45,48]. The expression and oligomerization status of the MojV and CedV sF constructs were confirmed by Western blot analysis (Figure S1B) and protein cross-linking assays (Figure S1C). Using purified MojV and CedV sF trimers, the HR2 heptad peptide triggering and capture assay was conducted. Similar to HeV and NiV sF constructs, the MojV and CedV sF are expressed as predominantly unprocessed trimeric F_0_ precursors (Figure S1B and C), but batch to batch sF preparations can exhibit some cleavage into the F_1_/F_2_ subunits. To mimic the proteolytic processing of the F_0_ precursor into its F_1_ and F_2_ subunits, MojV and CedV sF were digested with trypsin and heat was used to trigger their pre- to post-fusion conformational change. The sF trimers were treated with various orders of application of trypsin and heat in presence and absence of their respective FC2 peptides, followed by avidin agarose bead precipitation and SDS-PAGE and Western blot analysis (Figure 7).

Similar to the findings reported for HeV and NiV sF [45], a combination of trypsin followed by addition of FC2 peptide then followed by heat application can capture a conformational intermediate of MojV and CedV sF as the trimer transitions from a pre-fusion to a post-fusion state. As was shown for NiV and HeV sF [45], it was noted that only trypsin-processed CedV sF could be triggered into this conformational transition by heat treatment and captured by the FC2 peptide (Figure 7B). Interestingly, however, MojV sF could also be captured after heat application in the presence of FC2 peptide without trypsin treatment (Figure 7B). Although this preparation of MojV sF trimer was apparently partially processed into the F_1_ and F_2_ subunits, it suggested that the pre-fusion conformation of the MojV sF might be less stable in comparison to other henipavirus sF trimers. An analysis of purified preparations of MojV and CedV sF oligomers by nsEM revealed that the glycoproteins were monodispersed trimers, but were primarily in post-fusion conformations when immobilized onto the grids (Figure S4B,D). Both pre-fusion and post-fusion conformations of trimeric MojV sF were identified (Figure S4B) and only post-fusion conformation of CedV sF was observed with a new preparation (Figure S4D). Although this could suggest some conformation instability of these sF species designed and prepared here, variations in the amounts of pre-fusion and post-fusion conformations of individual sF preparations do occur. We have also observed sF trimers of NiV and HeV fold to a post-fusion conformation upon freez–-thaw or when immobilized onto ELISA plates, as indicated from a loss of recognition by a pre-fusion specific mAb known as 5B3 [45,48]. We believe the heptad peptide triggering and capture assay using sF glycoproteins in solution is a reliable assessment of the pre- to post-fusion conformational change of a sF glycoprotein preparation.

### 3.7. The Low and Slow MojV F Mediated Fusion Phenotype Is Not Attributable to a Defect in Function

**Figure 8:**
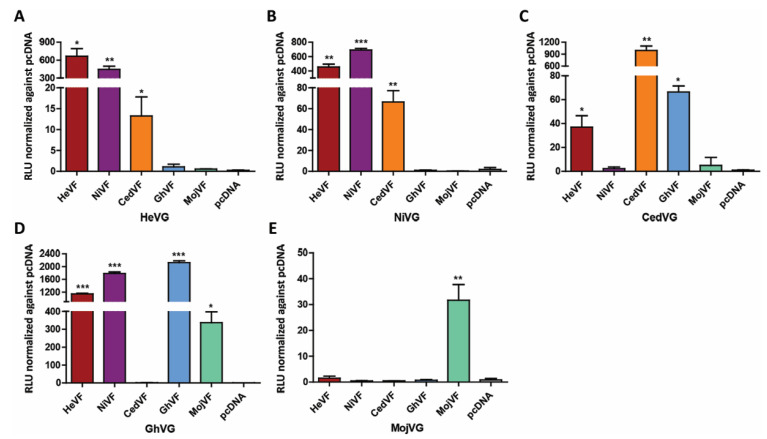
Heterotypic henipavirus F and G complementation assay confirms MojV F functionality in a quantitative Nano luciferase reporter cell–cell fusion assay. BSR-T7/5 effector cells were co-transfected with empty vector or henipavirus F and either (**A**) HeV G, (**B**) NiV G, (**C**) CedV G, (**D**) GhV G or (**E**) MojV G. Effector cells were co-incubated with C6 rat glial cells transfected with the pTM1-Nluc reporter plasmid. Maximum RLUs were recorded 48 h post-application of target cells to all effector monolayers, except for co-cultures with effector cells expressing MojV G, which reached maximum RLUs on day 4. Maximum RLUs were normalized against background defined as RLUs from mixtures of the target C6 cells applied to effector cells co-transfected with henipavirus G and empty vectors. The experiments were performed at least 3 times in technical triplicates. A representative experiment is shown here. Data were analyzed by Welch’s *t* test. The error bars represent the standard deviation. * *p* ≤ 0.05, ** *p* ≤ 0.01, *** *p* ≤ 0.001.

Co-expressed heterotypic combinations of HeV, NiV and GhV F and G have been shown to retain functionality and mediate cell fusion [60,62]. Here, the respective contribution of the MojV F and G glycoproteins to the cell fusion process was examined by heterotypic F/G glycoprotein cell fusion assays to further evaluate the MojV F glycoprotein fusion phenotype. Individual BSR-T7/5 effector cell populations were produced using each henipavirus F glycoprotein paired individually with each henipavirus G glycoprotein, and C6 rat glial cells were used as target cells since this cell line supported the highest levels of MojV-mediated cell fusion and was also permissive for CedV and HeV-mediated cell fusion. The Nano luciferase reporter gene cell–cell fusion assay was conducted simultaneously with all cell combinations (Figure 8).

Heterotypic combinations of HeV and NiV F and G were functionally competent in a bidirectional manner as previously reported [60]. In addition, HeV G and NiV G successfully triggered CedV F to mediate cell–cell fusion, albeit at lower levels than their homotypic pairings and the heterotypic HeV/NiV pairings (Figure 8A–C). Co-expression of NiV F with CedV G did not result in cell fusion, but the HeV F/CedV G paring was cell fusion competent. The CedV G/MojV F and the CedV F/MojV G pairings were not cell fusion competent. (Figure 8E). The MojV F and G-mediated cell fusion was again functional, but notably the MojV F and GhV G pairing was also cell fusion competent as previously reported (Figure 8D) [39]. However, the level of MojV F and GhV G-mediated cell fusion was increased 10-fold and did not display the slow cell fusion phenotype exhibited by the homotypic MojV F and G pairing-mediated cell fusion. Whereas the maximum fusion levels mediated by the homologous MojV F and G pairing were recorded on day 4, those mediated by the heterologous pairing MojV F/GhV G were observed at the 48 h time point. Together, with the earlier biochemical and functional analyses, these data indicate that the low and slow fusion phenotype of the MojV F and G glycoproteins is not due to a functional defect in the MojV F but more likely due to MojV G receptor restrictions.

## 4. Discussion

In the absence of an infectious isolate of MojV, which is known only from sequence data, details about the cellular and species tropism, receptor use and infection process, as well as any pathogenic potential of this henipavirus, remain quite limited. Over the past more than two decades, studies using henipavirus F and G glycoproteins, produced by recombinant techniques, as surrogate for infectious virus, have provided a wealth of information on the infection tropism and virus–host cell interactions of henipaviruses. Here, we investigated the functional and biochemical characteristics of the F and G glycoproteins of MojV. Using established techniques, we demonstrated that, although functional, MojV F and G-mediated cell fusion possessed a low and slow cell fusion phenotype when compared to HeV and CedV, which was in agreement with observations previously reported on MojV F and G-mediated cell fusion that was described as “significantly less robust” than cell fusion mediated by NiV-F and G [39]. In addition to confirming previous findings with human HEK293T and A549 and rodent BSR-T7/5 and BHK-21 cells [39], we found that MojV F and G-mediated cell fusion was permissive with several other cell lines of human (HeLa-USU), non-human primate (Vero) and rodent origin (L2, Rat2 and C6). An exhaustive analysis of potential ephrin ligand interaction with MojV G and comparison to that of CedV G, which was earlier shown capable of a functional interaction with mouse ephrin-A1 [31], found no significant binding interactions between MojV G and any A-class or B-class ephrin proteins tested in co-precipitation and BLI assays. Although we detected very low levels of apparently specific cell fusion mediated by MojV F and G glycoproteins with cells expressing mouse ephrin-A1, rat ephrin-A4 and human ephrin-A5, the low levels and slow kinetics of cell fusion do not support a conclusion that these proteins are natural entry receptors for MojV.

Potential biochemical or structural defects in the recombinant expressed MojV F and G glycoproteins that could account for the low and slow cell fusion phenotype observed here and in previous studies [39] were not identified. The data here showed that recombinant MojV F and G share similarities in their expression and migration patterns in comparison to HeV and CedV F and G, suggesting proper structure and folding, and recapitulated the higher order oligomerization status of F and G seen with other henipavirus F and G glycoproteins. In addition, an HR2 heptad peptide triggering and capture assay provided evidence that recombinant MojV sF is functionally competent with a similar pre- to post-fusion conformational transition capacity as previously observed with NiV and HeV sF [45,48] and with CedV sF shown here. Notably, however, MojV sF trimers, in apparently both pre- and post-fusion conformations, of predominantly unprocessed F_0_ subunits could be readily detected in the HR2 heptad peptide triggering and capture assay just by raising the temperature. Indeed, this observation did lead to an initial speculation that the observed low and slow cell fusion phenotype may be the result of having a significant proportion of the recombinantexpressed MojV F on the cell surface already in a post-fusion or partially triggered form. However, an analysis of the respective contribution of the MojV F and G glycoproteins to the cell fusion process revealed that MojV F facilitated significant cell fusion in the context of its triggering by GhV G, known to engage with ephrin-B2 as its functional receptor [30,38], in comparison to its homologous MojV G glycoprotein. Additionally, the possibility that the instability observed here with recombinant MojV sF is artificial cannot be excluded because the sF trimer is removed from the context of an intact virion. Indeed, work by Cifuentes-Muñoz et al. suggests that the HeV F and the virus matrix (M) proteins pre-assemble before they reach the plasma membrane [63]. Taken together, the findings presented here suggest that the low and slow cell fusion phenotype exhibited by the MojV F and G glycoproteins likely result from entry receptor restrictions rather than an inherent functional defect in MojV F. The possibility that recombinant MojV G encodes defects that prevent proper receptor engagement also cannot be ruled out, and such defects may be present owing to the discovery of MojV by genetic sequence data alone. The natural fusion-triggering and entry receptor for MojV may be expressed at levels too low, on the various cell lines tested in the present study, for robust cell fusion to be detected. Experiments here focused on examining the susceptibility of cells of primate and rodent origin to MojV F and G-mediated membrane fusion and while *Pteropus* bats are not found in China, non-*Pteropus* species such as bats of the genus *Rousettus* might be potential reservoirs for MojV. An examination of MojV F and G-mediated syncytia formation and fusion kinetics in non-*Pteropus* bat cells will be important. However, the results here do suggest that neither A- nor B-class ephrin ligands serve as primary functional receptors of MojV.

Nevertheless, the discovery and characterization of both CedV and MojV suggest the possibility of additional natural hosts for the genus *Henipavirus*. Indeed, although mice were initially reported as resistant to HeV and NiV infections [64,65], Dups et al. [66] established a model of HeV encephalitis in aged mice infected by intranasal inoculation. Further, the golden hamster is now a well-accepted model of NiV and HeV infection and pathogenesis [65,67]. Additionally, the A-class rodent ephrin ligand (mouse ephrin-A1) supports cell fusion and infection by CedV [31]. In addition, the present study shows that rat cells are susceptible to HeV, CedV and MojV F and G-mediated cell fusion, with rat C6 glial cells serving as a particularly competent fusion permissive cell line with MojV G and F glycoproteins. That rodents could serve as natural hosts or virus amplifiers of CedV and MojV therefore appears possible.

The type I INF response antagonists V and W proteins have been shown to play a critical role in NiV and HeV pathogenesis [68,69]. Analysis of the MojV genomic data reveals the RNA editing sites that would allow for the expression of MojV V and W proteins [17], which the non-pathogenic CedV lacks [69]. The pathogenic potential of MojV remains unknown at present, but could be explored by virus rescue using reverse genetics techniques under BSL-4 containment, and the present studies have provided some insight into the functional characteristics of the critically important MojV structural glycoproteins that could facilitate virus rescue studies.

## Data Availability

Not applicable.

## Supplementary Materials

The following are available online at https://www.mdpi.com/1999-4915/13/3/517/s1, Figure S1: Design, expression and oligomerization of MojV sF; Figure S2: Design, expression and oligomerization status of MojV and GhV sG glycoproteins, soluble rat ephrin-A4-Fc expression and protein/protein interaction assessment by co-precipitation and biolayer interferometry assays; Figure S3: Size exclusion chromatography, blue native (BN) PAGE and Western blot analyses of soluble henipavirus G and F glycoproteins; Figure S4: Electron microscopy analysis of MojV and CedV sF, and GhV and MojV sG glycoproteins; Figure S5: Syncytia formation is not detected in MojV F and G transfected Vero and HEK293T cells by microscopy.
