# Supplementary material for: Functional Analysis of the Fusion and Attachment Glycoproteins of Mojiang Henipavirus
Source: Viruses. 2021 Mar 22;13(3):517. doi: 10.3390/v13030517 (PMC8004131; doi:10.3390/v13030517)
Supplement: Supplementary file 1 [file viruses-13-00517-s001.pdf]

Supplementary figures: Cheliout Da Silva, S.; Yan, L.; Dang, H.V.; Xu, K.; Epstein, J.H.; Veesler, D.; Broder, C.C. Functional Analysis of the Fusion and Attachment Glycoproteins of Mojiang Henipavirus. *Viruses* **2021**

Figure S1

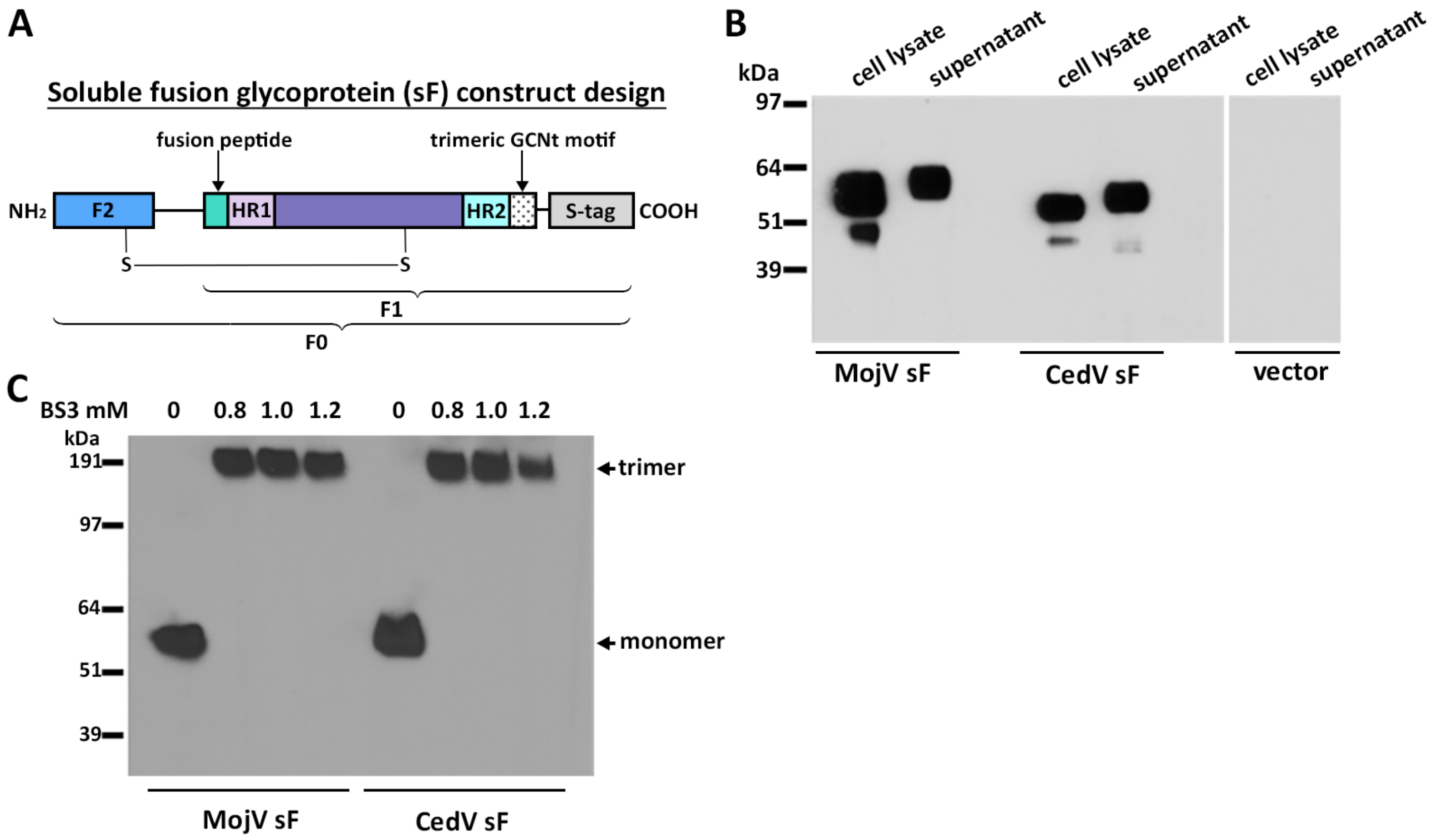

Supplementary Figure S1. Design, expression, and oligomerization of MojV and CedV sF glycoproteins. **(A)** The constructs of recombinant MojV and CedV soluble fusion (sF) glycoproteins were designed to include a GCN4 motif (MKQIEDKIEEILSKIYHIENEIARIKKLIGE) (GCNt) to promote trimerization of sF glycoproteins into the native conformational state adopted by cell-surface expressed henipavirus F glycoproteins. **(B)** Transient expression of recombinant MojV sF and CedV sF glycoproteins was confirmed in HEK293T cells. Cell lysates were precipitated 48 h post transfection with S agarose beads and analyzed by SDS-PAGE and western blot with polyclonal anti-S:HRP antibody. **(C)** To assess their oligomerization status, purified MojV and CedV sF glycoproteins were treated with increasing concentrations of cross-linking reagent BS3 (0, 0.8, 1.0, 1.2 mM). The oligomers (50 ng) were subjected to western blot analysis under non-reducing conditions with detection by polyclonal anti-S:HRP antibody.

Figure S2

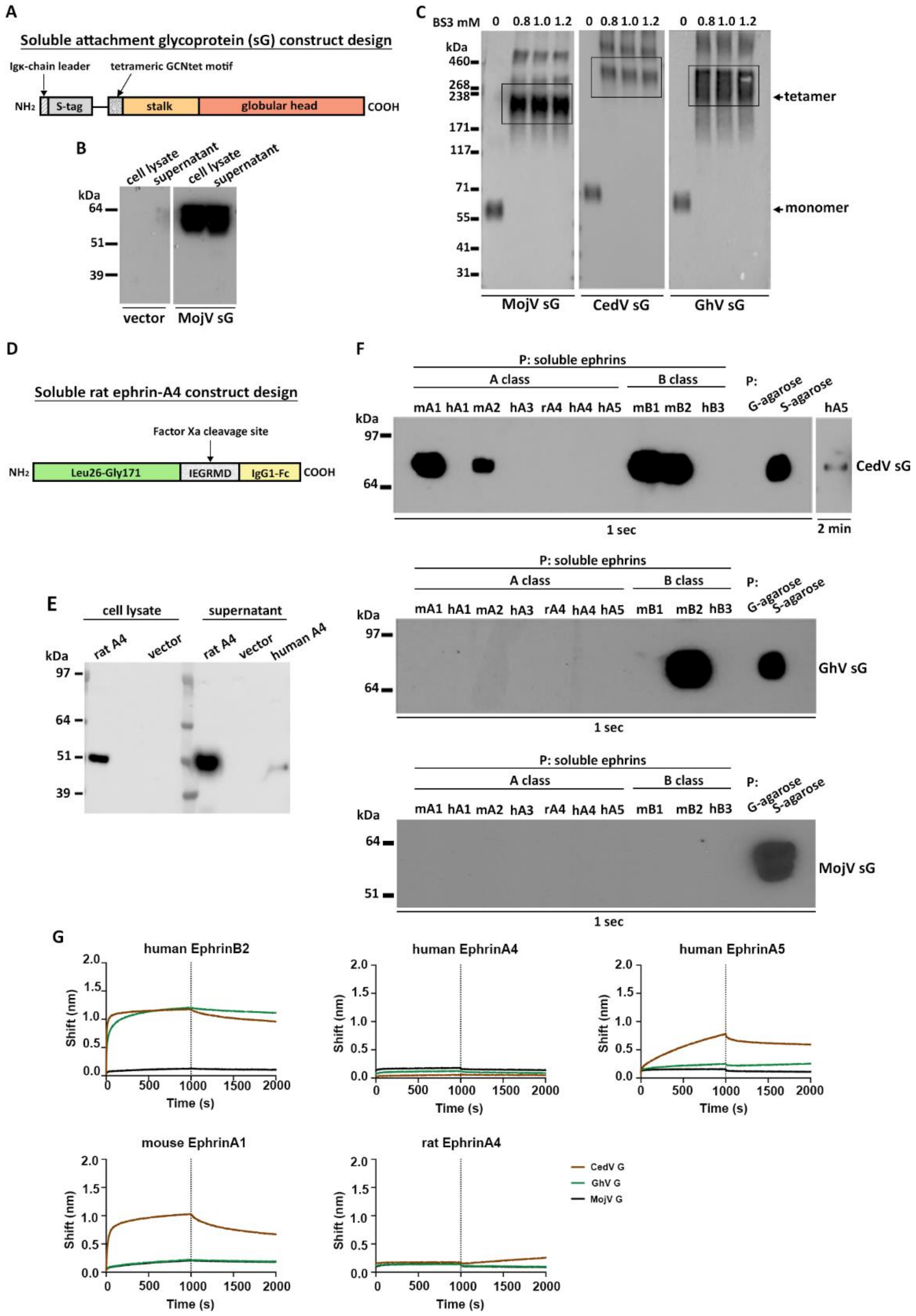
A
Soluble attachment glycoprotein (sG) construct design
Igκ-chain leader
tetrameric GCNtet motif
NH2
S-tag
stalk
globular head
COOH
B
cell lysate
supernatant
cell lysate
supernatant
kDa
64
51
39
vector
MojV sG
C
BS3 mM
0
0.8
1.0
1.2
kDa
460
268
238
171
117
71
55
41
31
tetamer
monomer
MojV sG
CedV sG
GhV sG
D
Soluble rat ephrin-A4 construct design
Factor Xa cleavage site
NH2
Leu26-Gly171
IEGRMD
IgG1-Fc
COOH
E
cell lysate
supernatant
rat A4
vector
rat A4
vector
human A4
kDa
97
64
51
39
F
P: soluble ephrins
A class
B class
P:
G-agarose
S-agarose
mA1 hA1 mA2 hA3 rA4 hA4 hA5 mB1 mB2 hB3
hA5
kDa
97
64
CedV sG
1 sec
2 min
GhV sG
1 sec
MojV sG
51
1 sec
G
human EphrinB2
human EphrinA4
human EphrinA5
mouse EphrinA1
rat EphrinA4
Shift (nm)
Time (s)
0.0
0.5
1.0
1.5
2.0
0
500
1000
1500
2000
CedV G
GhV G
MojV G

Supplementary Figure S2. Design, expression and oligomerization status of MojV and GhV sG glycoproteins, soluble rat ephrin-A4-Fc expression and protein/protein interaction assessment by co-precipitation and biolayer interferometry assays. **(A)** Constructs of recombinant MojV and GhV soluble attachment (sG) glycoproteins were designed to include a GCN motif (MKQIEDKLEEIESKLKKIENELARIKK) (GCNtet) to allow tetramerization of the proteins. **(B)** The transient expression of recombinant MojV sG glycoprotein was confirmed in L2 rat cells. Cell lysates were precipitated 48 h post transfection with S agarose beads and analyzed by SDS-PAGE and western blot with polyclonal anti-S:HRP antibody. **(C)** The tetrameric oligomerization of MojV, and GhV sG and CedV sG [28] glycoproteins was tested by treating purified MojV, GhV sG and CedV sG glycoproteins with increasing concentrations of cross-linking reagent BS3 (0, 0.8, 1.0, 1.2 mM). The oligomers (50 ng) were analyzed by SDS-PAGE followed by western blot under reducing conditions and detected with polyclonal anti-S:HRP antibody. **(D)** A construct to express recombinant rat soluble ephrin-A4 protein was designed with an Fc tag at its carboxylic end. Clones were confirmed by sequencing (GeneWiz, Frederick, MD). **(E)** The transient expression of recombinant rat soluble ephrin-A4-Fc protein was tested in BSR-T7/5 cells. Human soluble ephrin-A4-Fc (R&D systems, Minneapolis, MN) was included as a control. Cell lysates were precipitated 48 h post transfection with G agarose beads and analyzed by SDS-PAGE and western blot using HRP conjugated-anti-human IgG (1:12,500). **(F)** A panel of soluble Fc-tagged A- and B-class ephrin ligands including mouse ephrin-A1, human ephrin-A1, mouse ephrin-A2, human ephrin-A3, rat ephrin-A4, human ephrin A4, human ephrin-A5, mouse ephrin-B1, mouse ephrin-B2 and human ephrin-B3, was co-precipitated with CedV, GhV or MojV sG glycoproteins and G agarose beads. MojV, CedV or GhV sG incubated with G agarose beads or S agarose beads in absence of ephrins were included as controls. Western blot analysis was performed to assess the interaction of MojV, CedV and GhV sG with each ephrin. The blots were probed with polyclonal anti-S:HRP antibody. No interactions were detected between MojV sG and the ephrin tested. **(G)** The binding of human ephrin-B2, -A4, -A5, mouse ephrin-A1 and rat ephrin-A4 to immobilized CedV, GhV and MojV sG was analyzed by biolayer interferometry.

Figure S3

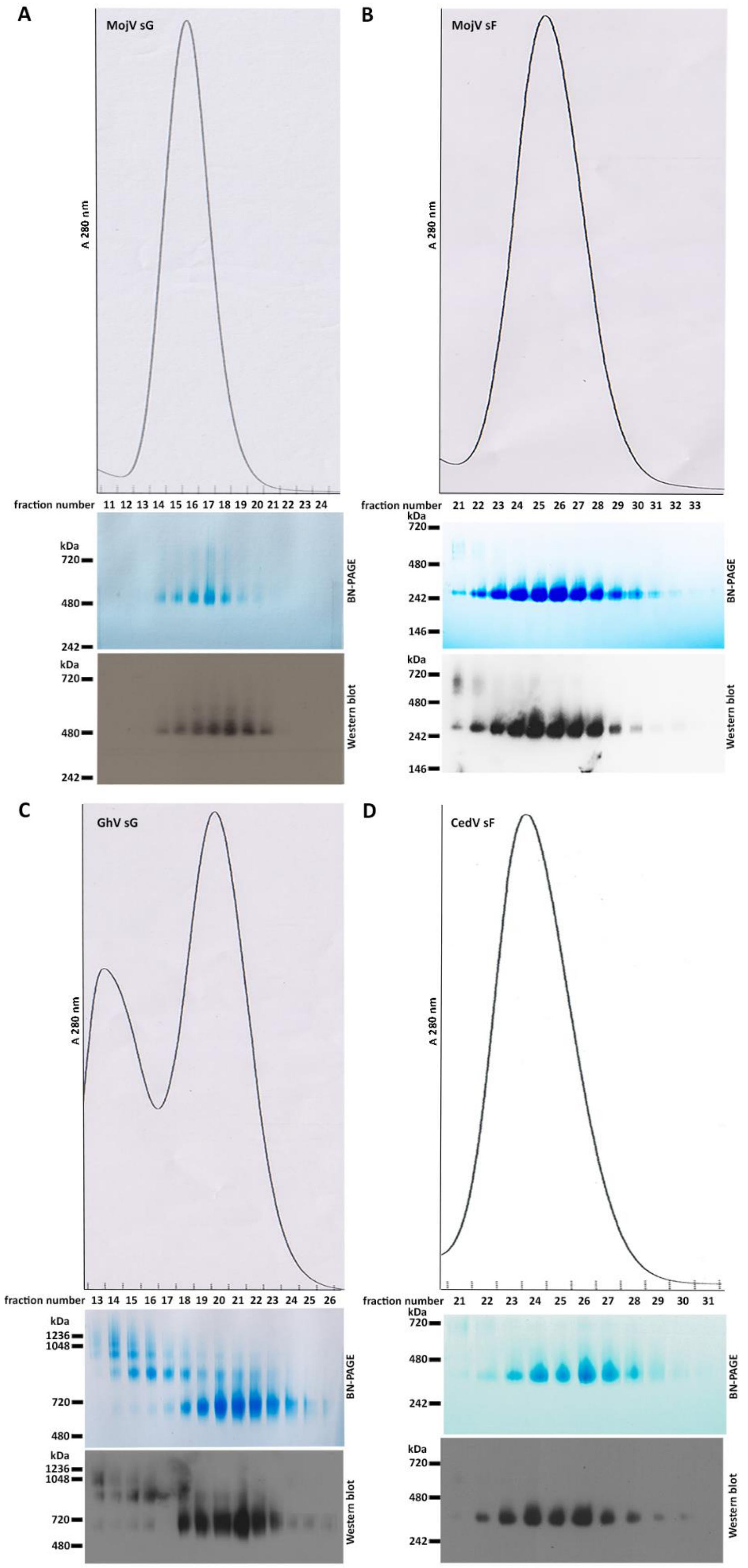

Supplementary Figure S3. Size exclusion chromatography, blue native (BN) PAGE and western blot analyses of soluble henipavirus G and F glycoproteins. (**A**) MojV sG glycoprotein was purified from Neuro-2a stable cell line supernatant, whereas (**B**) MojV sF (**C**) GhV sG and (**D**) CedV sF glycoproteins were purified FreeStyle™ 293 stable cell lines supernatants. Gel filtration profiles were obtained as previously described [39,42] by applying 2mg of affinity-purified protein to a HiLoad 16/60 Superdex 200 prep grade gel filtration column XK 16 (top panels). Out of each fraction collected 10 µl was analyzed by BN-PAGE followed by Coomassie Brilliant Blue stain (middle panels), and 1 µl by western blot detection with HRP-conjugated anti-S polyclonal rabbit antibody (bottom panels). MojV sG elution fractions 14-21 (**A**), MojV sF elution fractions 23-29 (**B**), GhV sG elution fractions 19-24 (**C**) and CedV sF elution fractions 22-29 (**D**) were pooled and concentrated for downstream applications.

Figure S4

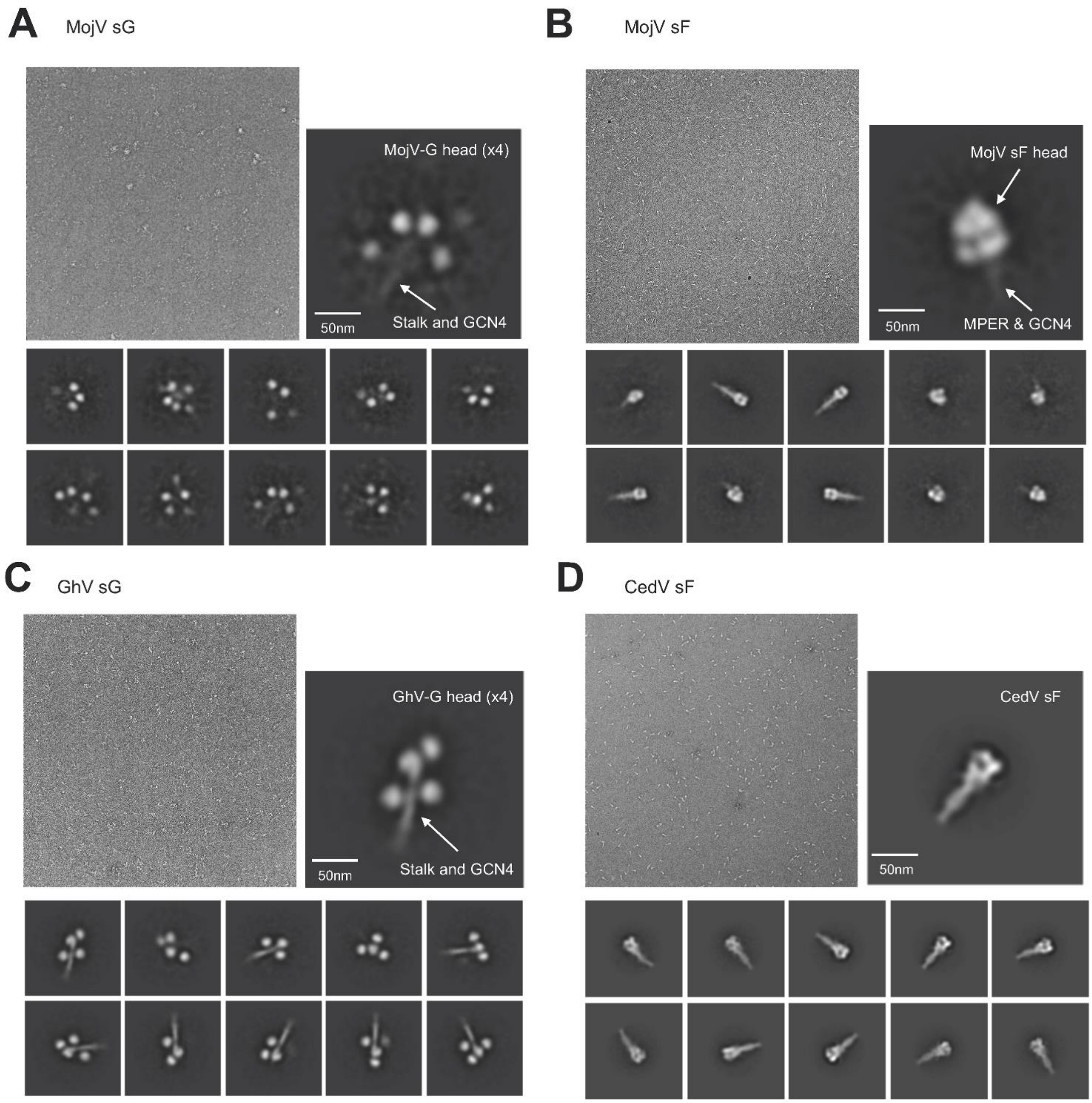

Supplementary Figure S4. Electron microscopy analysis of MojV and CedV sF, and GhV and MojV sG glycoproteins (**A**) MojV sG: A raw nsEM micrograph, 2D classification and a representative class showing head, stalk and GCN4 regions. (**B**) MojV sF: A raw nsEM micrograph, 2D classification (both pre-fusion and post-fusion forms) and a select class in tree shape (pre-fusion) showing membrane proximal external region (MPER) and GCN4 regions. (**C**) GhV sG: A raw nsEM micrograph, 2D classification and a representative class showing head, stalk and GCN4 regions. (**D**) CedV sF: A raw nsEM micrograph, 2D classification and a representative class in golf tee shape (post-fusion).

Figure S5

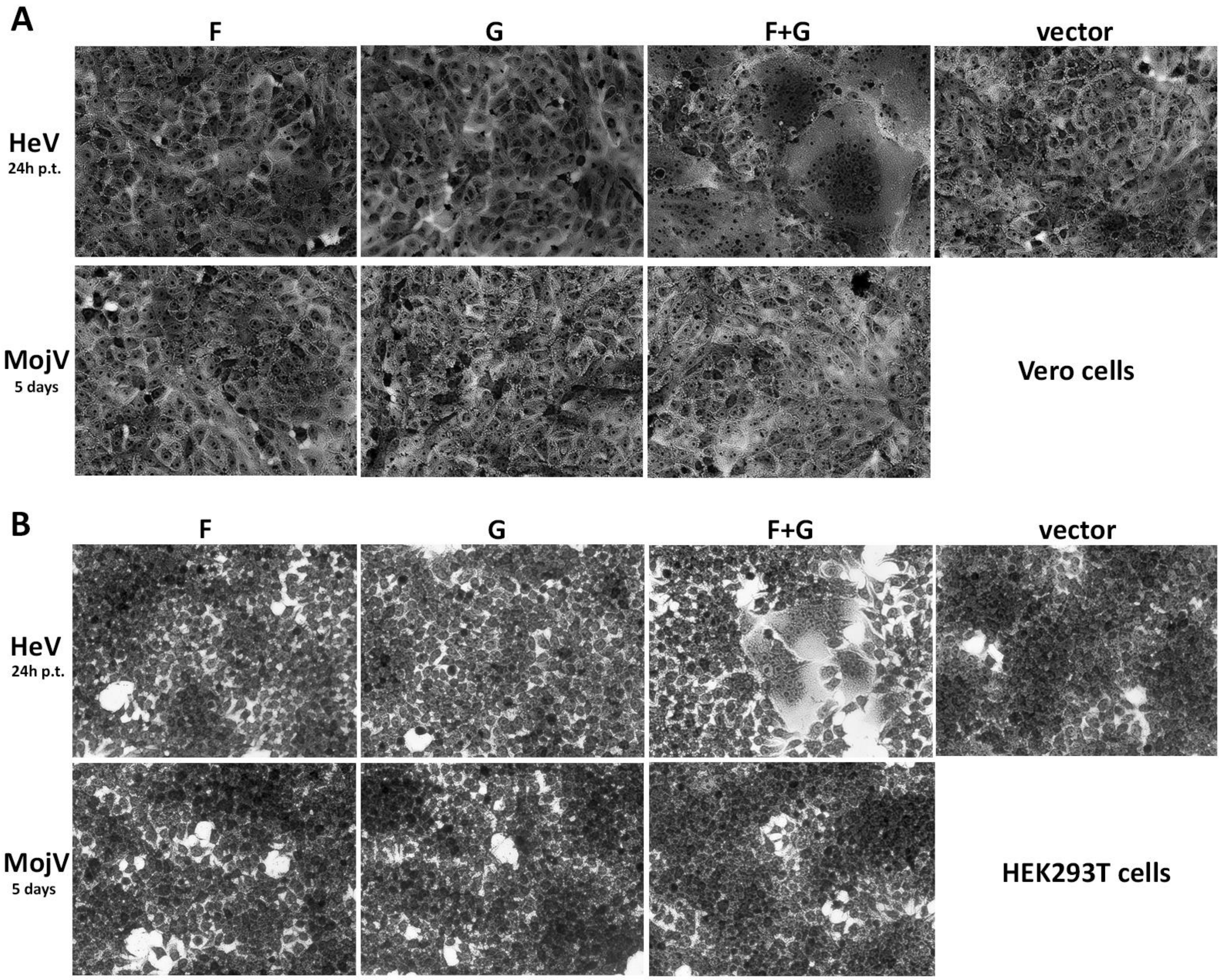

Supplementary Figure S5. Syncytia formation is not detected in MojV F and G transfected Vero and HEK293T cells by microscopy**. (A)** Vero and **(B)** HEK293T cells were transfected with pcDNA 3.1 Hygro CMV henipavirus F or G alone, or co-transfected with henipavirus F and G, or with empty vector. Upon observation of giant multinucleated cell formations (syncytia) the cells were fixed in methanol and stained with crystal violet at the indicated time. Images were obtained with a Zeiss Axio Observer A1 inverted microscope with a 20X objective. p.t.: post-transfection.
